# From the Difficult Airway Management to Diagnosis of Retropharyngeal Synovial Cell Carcinoma

**DOI:** 10.3390/children9091361

**Published:** 2022-09-07

**Authors:** Joanna Maria Jassem-Bobowicz, Ewa Magdalena Sokołowska, Katarzyna Monika Hinca, Izabela Drążkowska, Katarzyna Stefańska

**Affiliations:** 1 Division of Neonatology, Medical University of Gdańsk, 80-210 Gdańsk, Poland; 2 Scientific Students’ Circle, Division of Neonatology, Medical University of Gdańsk, 80-210 Gdańsk, Poland; 3 Division of Gynecology and Obstetrics, Medical University of Gdańsk, 80-210 Gdańsk, Poland

**Keywords:** newborn, polyhydramnios, airway patency, respiratory insufficiency, neonatal resuscitation, biphasic synovial sarcoma

## Abstract

Respiratory complications are among the most common problems addressed in neonatology in the first hours after birth, whereas the risk of any cancer in the neonatal period is 28 per million. Sarcomas, malignant mesenchymal neoplasms, account for about 8% of all neoplasms in the neonatal period. We report on a male neonate born at 36 + 4/7 weeks of gestation, diagnosed with retropharyngeal synovial carcinoma. Ineffective respiratory movements and generalized cyanosis were the first symptoms to be noted. On the ultrasound examination of the neck, a tumor of the retropharyngeal space was exposed, then visualized by an MRI of the head and neck. The biopsy analysis revealed the diagnosis of an extremely rare tumor in a neonate. The location of its growth was atypical, contributing to a diagnostic challenge. The neoplasm was treated solely with chemotherapy concordantly with the CWS protocol, individually customized for our patient. Preterm birth, as in our case, 36 + 4/7 weeks of gestation, may imply a possible need for resuscitation or support in the transition period. Aggressive high-grade tumors of the head and neck region are locally invasive and prone to metastasize. However, prognosis in infants is hard to estimate, therefore both individualized treatment and multidisciplinary care should be tailored to the needs of the patient.

## 1. Introduction

Pediatric head and neck tumors account for 3% to 5% of all tumors. Identification of such malignancies is highly important, as each requires a different management approach, further affecting its outcome. Neonatal soft-tissue tumors constitute 25% of all neoplasia occurring in infants, with 15% being malignant. Sarcomatoid carcinoma is a subtype of squamous cell carcinoma (SCC) and mainly hosts the upper aero-digestive tract. SCC accounts for almost 4% of all squamous cell carcinomas of the head and neck region in patients younger than 40 years old [1]. According to Global Cancer Observatory (GLOBOCAN) it is anticipated for the prevalence of HNSCC to increase by 30% in the adult population, yet no exact data have been estimated for neonates and younger children [2]. Knowledge of its occurrence is based mainly on isolated cases presented in the literature; therefore, we treat it as an extremely rare entity. This disparity suggests the pathogenesis varies with age, while simultaneously differs in etiological factors, clinical manifestation, and final outcomes. The management of those tumors is challenging, especially among neonates, as there are no specifically designed protocols or a convincing theory, supporting treatment establishments. 

Concurrently, the most common problems addressed in neonatology are respiratory complications in the first hours after birth. In near-term neonates, the main cause includes transient tachypnea of the newborn, respiratory distress syndrome, or pneumonia. However, other rare origins may also appear directly after birth, significantly, respiratory insufficiency may be caused by the mechanical obstruction of the respiratory tract, potentially a cancerous mass. Tumors arising from retropharyngeal location tend to be asymptomatic, which impedes prenatal diagnosis. Polyhydramnosis is one of the potential findings seen in the ultrasound examination, yet it is not pathognomonic.

“*What used to define a good neonatologist decades ago, was an ability to intubate any baby and now, after thirty years of my career, what defines a good neonatologist is the knowledge and experience to know when not to intubate*”

—*Neil Finer*

## 2. Case Presentation

**Figure 1:**
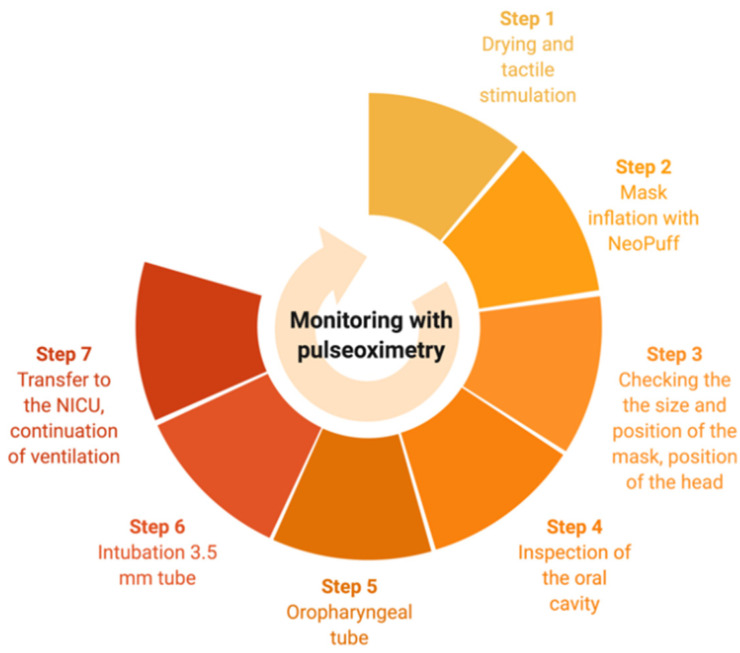
A timeline of actions performed to achieve airway control in the delivery room.

We present a male neonate delivered at 36 + 4/7 weeks of gestation with a birth weight of 2990 g by cesarean section due to the risk of asphyxia (episodes of bradycardia of the fetus after labor induction). The pregnancy was complicated by polyhydramniosis, hypothyroidism of the mother, and nine days of preterm premature rupture of membranes. The boy presented with good heart rate (140/min) and normal muscle tone. However, breathing was not effective and reaction to stimuli with generalized cyanosis and effort to breathe seen as abdominal muscle contractions were noted. In accordance with the European Resuscitation Guidelines, after umbilical cord clamping, tactile stimulation and drying were performed with no effect [3]. In the next step, mask inflation with NeoPuff^®^ was started (FiO2 0.21, PIP 30 cm H_2_O), however, chest movements were not observed, and the heart rate decreased to 90/min with blood saturation at a level of 50%. We checked for the position of the mask, its size, and head alignment. With no effect following these interventions, oral cavity inspection with the use of a laryngoscope was performed which allowed for visualization of the posterior wall of the pharynx. Primarily, the anatomy looked disturbed, the palate was lowered, and the pharynx seemed to be shallow. However, after the introduction of the laryngoscope, the baby took his first breath on his own. We, therefore. planned to keep the airway open with the introduction of the oropharyngeal tube. The size was measured from the angle of the mouth to the tip of the ear and introduced without twisting (a neonatal technique for oropharyngeal tube insertion). This procedure however impaired again the patency of the airway. With the deteriorating state of the neonate, a decision to intubate with a 3.5 mm neonatal tube was made. As it was a life-threatening situation and the baby commenced losing consciousness, there was no premedication before the procedure. Despite the fact that in direct laryngoscopy, the pharynx seemed to have a different axis and to be shallower than usual, there were no difficulties regarding the insertion of the tracheal tube. The depth of intubation was calculated according to the rule “6+weight in kilograms”. In our case, the estimated weight was 3 kg (the exact measurement was made after having stabilized the baby) and the depth of intubation was 9 cm measured at the mouth. The tube was then fixed with a medical patch. Equipment used for intubation consisted of a laryngoscope with Miller’s blade size “0” and a cuffless endotracheal tube. Intubation has been performed by a neonatal specialist who had a shift on that day. On the first attempt, the procedure was successful, and the baby got then transferred to the NICU to continue intensive care and to expand the diagnostic measures. The airway-control process is presented in Figure 1.

## 3. Diagnostic Process and Treatment

At admission to the NICU, the vital signs were within the normal range for neonates, values as follows: respiratory rate 40/min, heart rate 142/min, oxygen saturation 99%, arterial blood pressure 52/22 mmHg, temporal temperature 35.9 C degrees. Arteriole blood gases parameters were pH 7.36, pCO_2_ 40.2 mmHg, pO_2_ 47.6, BE–2.6 mmol/L. Following the admission, a chest X-ray was performed. The lungs were hyperinflated, otherwise, the image was normal (Figure S1). On the ultrasound examination of the neck, a tumor of the retropharyngeal space was exposed. After a thorough consult and inspection by consulting ENT specialist an abnormal mass was visualized with suspicion of neoplastic origin. An MRI of the head and neck was scheduled in order to precisely define where the original tumor was formed and then use it as further guidance for the excision procedure. A lesion originating from the palatine tonsil sized 27 × 22 × 19 mm was detected (Figure S2). After a pediatric oncologic consult, the incisional biopsy was performed by excising only a sample of the tumor for pathology examination. The surgical procedure was carried out under general anesthesia. 

The tumor was identified as biphasic synovial sarcoma. After having the incisional biopsy, further laboratory tests were conducted. Cytogenetic test by polymerase chain reaction detected the presence of the X;18 translocation, characteristic of the synovial sarcoma subtype. This chromosome mutation has been detected in samples of more than 90% os SSs and it has been suspected to impact the behavior of the neoplasm and its long-term prognosis. In our patient, the location site was not as it is typically expected. Synovial sarcomas tend to arise from the soft tissues of the para-articular regions and less commonly affect the head and neck area. After having successful partial removal of the tumor following the Cooperative Weichteilsarkom Studiengruppe protocol (CWS) the adjuvant chemotherapy was implied. On the 15th day of life, chemotherapy with vincrystyne, actinomycin D, and cyclophosphamide (VAC) adapted to the neonate’s weight and age was introduced off-label [4]. The main side-effects included repugnance. The patient was intubated since admission to the NICU, though after having started chemotherapy, he presented with nausea. Other gastrointestinal causes including infection of the gastrointestinal tract were excluded. The symptom was successfully treated with ondansetron. After 51 days from the beginning of the treatment, the mass decreased in size, and the baby was extubated. Four days later during cough, the infant expectorated a piece of greyish matter which on microscopic examination turned out to be the cells of the tumor mass that underwent necrobiosis. MRI performed afterward showed a complete regression of the neoplasm. After completion of the physiotherapy and gaining adequate sucking abilities, the boy was discharged home. Until now, he has undergone 21 hospital stays and 30 out-patient visits (some of which were telephone call follow-up visits). He is now in good shape and developing concordantly with his age (he is now 2 ^5^/_12_ years old). After having been discharged, he did not require any further interventions within the oral cavity.

## 4. Discussion

The most common immediate complications in near-term newborns are hypothermia, sepsis, hypoglycemia, respiratory distress, and hyperbilirubinemia [5]. Preterm birth itself may also carry a twelve-fold increase in the risk of resuscitation at birth [6]. According to European Resuscitation Council (ERC) guidelines, preterm birth, especially before 35 weeks of gestation may require either resuscitation or support in the transition period [3]. Following those guidelines, if the neonate does not breathe spontaneously at birth, first drying with tactile stimulation, then proper airway management (A—airway) and lung inflation/ventilation (B—breathing) should be performed. This should be confirmed by assessing the chest movement. In our patient, chest elevation was not observed. Therefore, the next steps in airway management had to be taken, ensuring the proper head position, then choosing the right size of face mask warranting no air leak. When this turns ineffective, the next step may be either a two-person mask application with subsequent ventilation or oropharyngeal mask use with inspection of the oral cavity and posterior wall of the pharynx. On the laryngoscopy, abnormal anatomy was noted. Due to visible shortness of breath, a decision to support the airway with an oropharyngeal mask was made. When this appeared ineffective a rapid decision to intubate was taken which resulted in adequate airway patency. Despite the fact, that intubation stands as the last means to achieve the proper airway, this case confirms that a full preparation is needed also for relatively low-risk deliveries and that the attending staff should be skilled in neonatal intubation. According to resuscitation guidelines worldwide, during low-risk deliveries, there should be at least one member of staff qualified in performing the first steps of resuscitation and positive pressure ventilation [7,8,9,10]. Management of neonatal respiratory impediments is substantial. However, due to an increasing tendency to apply non-invasive ventilation in neonates, even in the population of extremely preterm infants, the skills mandatory for a successful procedure are not being used and trained as often as they used to be decades ago. As a consequence, there is a need for continuous training of staff to ensure proper skills in rare situations when emergency intubation needs to be performed ad hoc. The proposed algorithm is regular training in a simulated environment [11,12,13], training on a cadaver, or during short anesthesia procedures.

The risk of any cancer in the neonatal period is 28 per million. Sarcomas account for about 8% of all neoplasms in the neonatal period, which makes them an extremely rare condition in that group [14]. Therefore, the diagnosis is not only challenging but also may come as an unexpected occurrence during routine neonatal management. Soft tissue sarcomas are not commonly known to appear in this certain location site. The neonatal neoplasm mostly occurring in this area is a hemangioma, a benign vascular tumor, usually of transient character, affecting 4% to 5% of infants. Deep hemangiomas are the ones forming in the areas of excess fatty tissue such as the neck region. At times, primarily, these might be confused and misdiagnosed with other soft tissue tumors. Therefore, prenatal diagnosis plays a critical role in advancing therapeutic approaches. For sarcomas, as for the para-articular regions of the limbs, the prenatal ultrasound might result in early detection of the tumor. Conversely, in our case, the location site constituted an impediment for the tumor to be visualized. Cancerous masses arising in the head and neck region may obstruct both the respiratory and digestive tract resulting in polyhydramniosis or lung hypoplasia. Therefore, we should consider tumors as one of the possible causes of greater amniotic fluid volume or intrauterine growth retardation. To our knowledge, no prenatal invasive methods were shown to increase the percentage of early detection of soft-tissue sarcomas. 

Most babies diagnosed with pediatric tumors require prolonged hospital stay due to the need for treatment administration, proper monitoring, and diagnostic procedures. In this case, because of respiratory insufficiency due to a large mass in the oral cavity, the first period of treatment required admission to the Neonatal Intensive Care Unit. On the other hand, the oncologic treatment itself must always be carried out by a pediatric oncologist. In our case, there were more than ten members of hospital staff involved in the treatment, namely a neonatologist, a pediatric oncologist, a pediatrician, an anesthesiologist, a radiologist, a pathologist, an ENT specialist, a nurse, a midwife, a psychologist, a physiotherapist, and a speech therapist. As soft tissue sarcomas can be difficult to diagnose, an experienced pathologist should always be involved in the analysis of the samples. However, the doctors leading direct care over the patient during the daily shifts must be acquainted with alarming symptoms in the course of oncologic treatment and cultivate close cooperation between the members of staff. The care must be individually tailored to meet the needs of the infant. Due to the rare prevalence of neonatal biphasic synovial sarcoma, there were no protocols available and a CWS protocol treatment had to be adapted for individual use. Similar challenges have been described earlier [15].

The patient presented in our case report did not require surgical treatment due to effective chemotherapy which resulted in tumor necrosis and its extruding. However, provided the patient needed surgery, due to small size of airways, access to operate through the mouth can be challenging. There have been very limited publications on transoral robotic access for head and neck cancers, however with the use of this method, the rate of complications is low, and the procedure seems to be safe [16].

Until now the patient is relapse-free, however, the prognosis is difficult to estimate due to the low number of studied cases. According to the literature, prognosis depends mainly on the tumor size, depth, invasion, and primary location in the pediatric population [17].

## 5. Conclusions

In this report, we presented a rare case of a head tumor in a neonate. This is to raise awareness of the fact that thorough following of the resuscitation guidelines in airway management is needed and in rare cases, tracheal intubation may be needed even in late preterm infants.

We emphasize the fact that neonatal neoplasms are extremely sparse, therefore an individualized treatment must be tailored to assure survival without long-term consequences. 

Prognosis in neoplastic treatment in infants is hard to estimate, therefore multidisciplinary care and parental counseling should be offered. 

## Data Availability

Not applicable.

## Supplementary Materials

The following supporting information can be downloaded at: https://www.mdpi.com/article/10.3390/children9091361/s1, Figure S1: chest X-ray after admission to the NICU; Figure S2: MRI scan of the neck, arrow pointing at the tumor.
