# Supplementary material for: From the Difficult Airway Management to Diagnosis of Retropharyngeal Synovial Cell Carcinoma
Source: Children (Basel). 2022 Sep 7;9(9):1361. doi: 10.3390/children9091361 (PMC9498277; doi:10.3390/children9091361)

Figure S1. Chest X-ray after admission to the NICU.

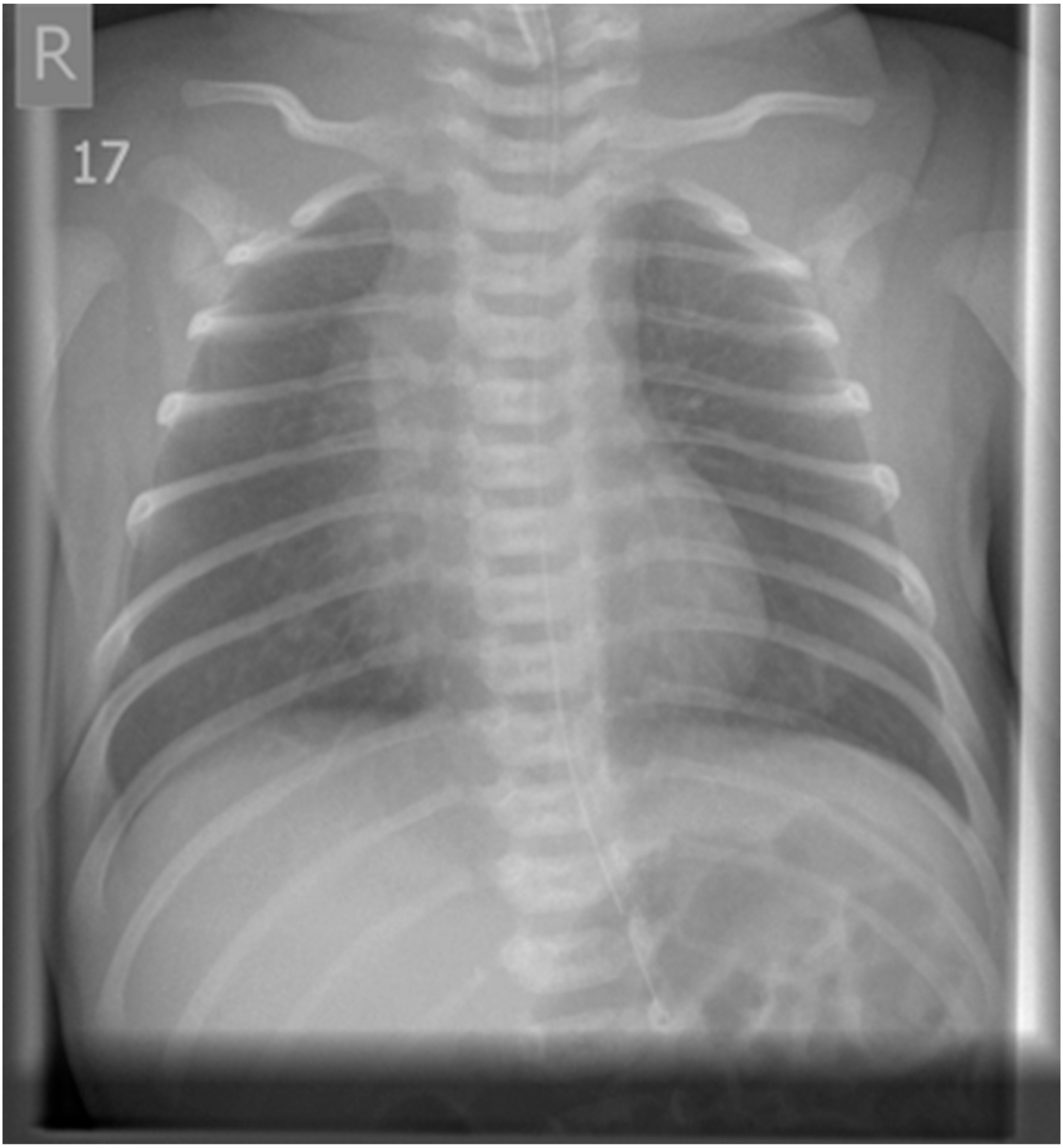

Figure S2. MRI scan of the neck, arrow pointing at the tumor.

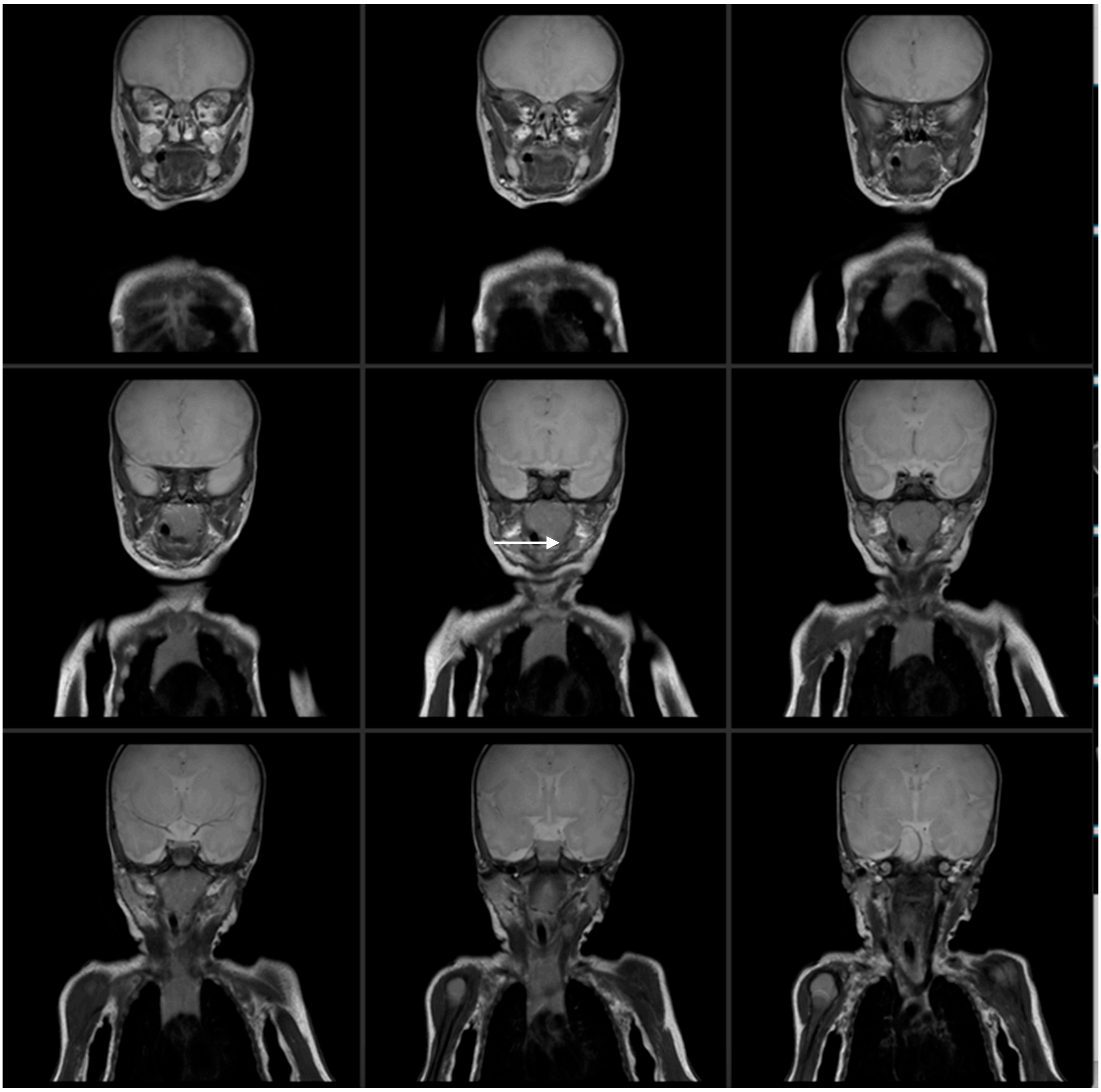

Supplement: Supplementary file 1 [file children-09-01361-s001.zip › children-1734312-supplementary.pdf]
